# A Non Invasive Estimate of Dead Space Ventilation from Exercise Measurements

**DOI:** 10.1371/journal.pone.0087395

**Published:** 2014-01-30

**Authors:** Paola Gargiulo, Anna Apostolo, Pasquale Perrone-Filardi, Susanna Sciomer, Paolo Palange, Piergiuseppe Agostoni

**Affiliations:** 1 SDN Foundation, Institute of Diagnostic and Nuclear Development, Naples, Italy; 2 Centro Cardiologico Monzino, IRCCS, Milan, Italy; 3 Department of Advanced Biomedical Sciences, Division of Cardiology, “Federico II” University, Naples, Italy; 4 Department of Cardiovascular and Respiratory Sciences, “La Sapienza” University, Rome, Italy; 5 Department of Public Health and Infectious Diseases, Division of Pulmonary Research, “La Sapienza” University, Rome, Italy; 6 Department of Clinical Sciences and Community Health, Cardiovascular Section, University of Milan, Milan, Italy; 7 Department of Medicine, Division of Pulmonary and Critical Care Medicine, University of Washington, Seattle, Washington, United States of America; Virginia Commonwealth University, United States of America

## Abstract

**Rationale:**

During exercise, heart failure patients (HF) show an out-of-proportion ventilation increase, which in patients with COPD is blunted. When HF and COPD coexist, the ventilatory response to exercise is unpredictable.

**Objectives:**

We evaluated a human model of respiratory impairment in 10 COPD-free HF patients and in 10 healthy subjects, tested with a progressive workload exercise with different added dead space. We hypothesized that increased serial dead space upshifts the VE vs. VCO_2_ relationship and that the VE-axis intercept might be an index of dead space ventilation.

**Measurements:**

All participants performed a cardiopulmonary exercise test with 0, 250 and 500 mL of additional dead space. Since DS does not contribute to gas exchange, ventilation relative to dead space is ventilation at VCO_2_ = 0, i.e. VE-axis intercept. We compared dead space volume, estimated dividing VE-axis intercept by the intercept on respiratory rate axis of the respiratory rate vs. VCO_2_ relationship with standard method measured DS.

**Main results:**

In HF, adding dead space increased VE-axis intercept (+0 mL = 4.98±1.63 L; +250 mL = 9.69±2.91 L; +500 mL = 13.26±3.18 L; p<0.001) and upshifted the VE vs.VCO_2_ relationship, with a minor slope rise (+0 mL = 27±4 L; +250 = 28±5; +500 = 29±4; p<0.05). In healthy, adding dead space increased VE-axis intercept (+0 mL = 4.9±1.4 L; +250 = 9.3±2.4; +500 = 13.1±3.04; p<0.001) without slope changes. Measured and estimated dead space volumes were similar both in HF and healthy subjects.

**Conclusions:**

VE-axis intercept is related to dead space ventilation and dead space volume can be non-invasively estimated.

## Introduction

The behaviour of ventilation during exercise in heart failure (HF) and in chronic obstructive pulmonary disease (COPD) patients may differ, being characterized in the former by an out-of-proportion increase of ventilation (VE), which is greater the greater the HF severity [1] and, in the latter, by a normal or excessive increase of ventilation in mild or moderate COPD and a blunted ventilation increase in severe COPD patients [2]–[4]. The elevated ventilatory response in HF patients seen before lactic acidosis ensues and the carbon dioxide (CO_2_) [5] generated by the lactate is trivial relative to the rate of metabolic CO_2_ production (VCO_2_) [6], [7]. The relationship between VE and VCO_2_ is used to evaluate ventilatory efficiency [8]; in HF, as well as in pulmonary arterial hypertension, an increase of the slope of the VE vs. VCO_2_ relationship is associated with a poor prognosis [9]–[16]. In COPD, ventilatory limitation to exercise is defined either as a reduction of ventilatory reserve or as a lowering of inspiratory capacity [17]. In case of severe COPD, the rise of ventilation during exercise is blunted, and consequently the slope of VE vs. VCO_2_ relationship is normal or low, being the slope lower the more pronounced the emphysema profile [2].

HF and COPD often coexist with a reported prevalence of COPD in HF patients ranging between 23 and 30% [18] and with a relevant impact on mortality and hospitalization rates [19]. In patients with COPD and HF, the ventilatory response to exercise is poorly predictable. Indeed, HF hyperventilation can be counteracted by the incapacity of increasing tidal volume (VT) and alveolar ventilation, both being distinctive features of VE during exercise in COPD patients [17]. As a result, the slope of VE vs.VCO_2_ relationship might be elevated, normal or even low in patients with COPD and HF, regardless of the presence and of the severity of ventilatory inefficiency. Up to now, only few studies have evaluated the ventilatory behaviour during exercise in patients with coexisting HF and COPD, being patients with comorbidities usually excluded from research trials dedicated to HF or COPD [20].

In the present study, we evaluated HF patients and healthy individuals through a progressive workload exercise with different added DS, hoping to mimic at least in part the effects of COPD on ventilation behaviour during exercise. We hypothesized that increased serial DS upshifts the VE vs. VCO_2_ relationship and that the VE-axis intercept (VE_Yinter_) might be an index of DS ventilation. Indeed, since DS does not contribute to gas exchange, VE relative to DS is VE at VCO_2_ = 0, i.e., VE_Yint_ on the VE vs. VCO_2_ relationship.

## Methods

### Subjects

Ten HF patients and 10 healthy subjects were enrolled in the present study.

HF patients were regularly followed-up at our HF unit. Study inclusion criteria for HF patients were New York Heart Association functional classes (NYHA) I to III, echocardiographic evidence of reduced left ventricular systolic function (left ventricular ejection fraction ≤40%), optimized and individually tailored drug treatment, stable clinical conditions for at least 2 months, capability/willingness to perform a maximal or near maximal cardiopulmonary exercise test (CPET). Patients were excluded if they had obstructive and/or restrictive lung disease (forced expiratory volume in first second/forced vital capacity ratio (FEV_1_/FVC) <0.70% and/or lung vital capacity (VC) <80% of predicted value [21]), clinical history and/or documentation of pulmonary embolism, primary valvular heart disease, pulmonary artery hypertension, pericardial disease, exercise-induced angina, ST changes, severe arrhythmias and significant cerebrovascular, renal, hepatic and haematological disease.

A group of age matched healthy subjects was recruited among the hospital staff and from the local community through personal contacts. Inclusion criteria were absence of history and/or clinical evidence of any cardiovascular or pulmonary or systemic disease contraindicating the test or modifying the functional response to exercise, any condition requiring daily medications, and the inability to adequately perform the procedures required by the protocol. No subjects were involved in physical activities other than recreational.

The investigation was approved by the local ethics committee (“Ethics committee Centro Cardiologico Fondazione Monzino”, Institutional Review Board no. S186/311) and all participants signed a written informed consent before enrolling in the study.

### Study protocol

At enrolment, demographical and clinical data were collected, lung function measurements and echocardiographic evaluation were performed to verify that the subjects screened met the study inclusion/exclusion criteria, and the informed consent was obtained.

Spirometry (Vmax 29C, SensorMedics, Yorba Linda, CA, US) was performed by all participants in accordance with the recommended technique [22], and measurements were standardized as percentages of predicted normal values [23].

To become familiar with the procedure, both HF patients and healthy subjects had been previously trained to perform an exercise test in our laboratory [24]. Thereafter, on different days, following a random order, exercise testing was done with additional DS equal to 0 mL, 250 mL and 500 mL.

All participants underwent incremental CPET on an electronically braked cycle-ergometer (Ergometrics-800, SensorMedics, Yorba Linda, CA, US) using a personalized ramp protocol that was chosen aiming at a test duration of 10±2 minutes. The exercise was preceded by 5 minutes of rest gas exchange monitoring and by a 3-minute unloaded warm-up. A 12-lead ECG, blood pressure and heart rate were also recorded, and arterial oxygen saturation was monitored through a pulse oxymeter. The participants wore a nose clip and breathed through a mouthpiece connected to a mass flowmeter (Vmax 29C, SensorMedics, Yorba Linda, CA, US). Subjects were asked to cycle at a pedalling rate of 60–70 rpm, and CPET were self-terminated by the subjects when they claimed that maximal effort had been achieved. Oxygen consumption (VO_2_), VCO_2_ and VE were measured breath by breath with flowmeter and respiratory gas sampling lines at the end of the added DS. They were averaged every 20 seconds. Anaerobic threshold (AT) was calculated with the standard technique [25]. All tests were executed and evaluated by 2 expert readers.

**Figure 1:**
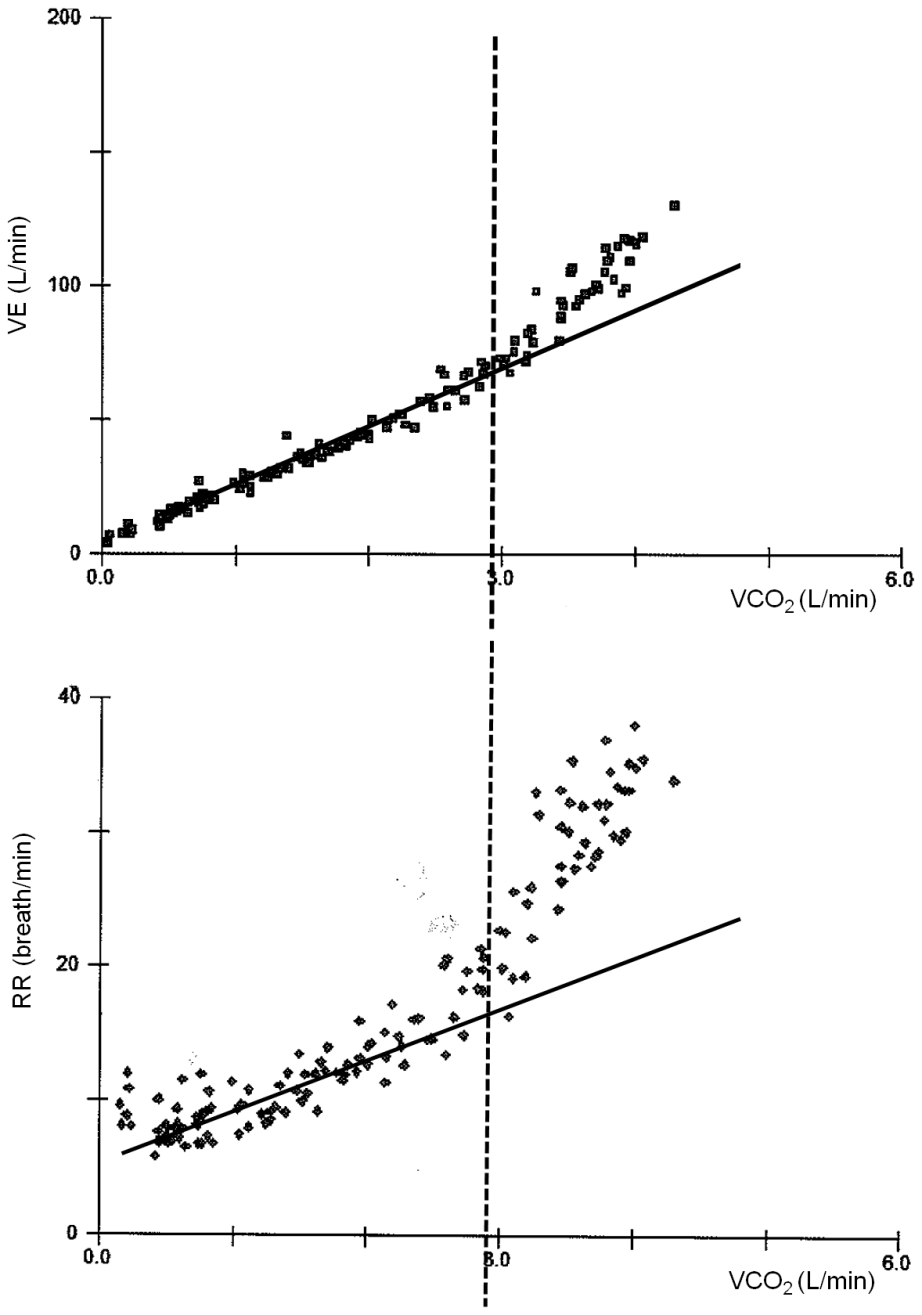
VE vs. VCO_2_ relationship in a patient. The relationship is linear up to the respiratory compensation point (end of the isocapnic buffering period) (Upper panel). RR vs. VCO2 relationship. The relationship is calculated as for VE vs. VCO_2_ (Lower panel).

In the absence of psychogenic hyperventilation, below the respiratory compensation point [26], the relation between VE and VCO_2_ is characterized by a linear relationship (VE = aVCO_2_+ b), with “a” as the slope and “b” as the intercept on the VE axis (VE_Yint_) [8]. Since DS does not contribute to gas exchange, it is possible to hypothesize that the ventilation relative to DS is similar or related to the VE at VCO_2_ = 0, which is the Y intercept of VE vs. VCO_2_ relationship. To calculate DS volume (VD) from VE_Yint_ (VD_Yint_), we need to identify the corresponding respiratory rate (RR). This was obtained as the intercept of the RR vs. VCO_2_ relationship on the RR axis (RR_Yint_). Specifically, the RR vs. VCO_2_ relationship was calculated through its linear portion that starts from the beginning of exercise and ends when RR increases more steeply, which corresponds to the tidal volume inflection/plateau [27], [28]. An example on how we calculate VE_Yint_ and RR_Yint_ is reported in figure 1.

We compared estimated VD values (VD_Yint_) with resting and exercise values of VD, measured with standard method [8] (VD_meas_), in the 3 experimental conditions, with 0 mL, 250 mL and 500 mL of added DS. The volume of mouthpiece and flowmeter (50 mL) was subtracted from VD. The standard calculation of VD [8] (VD_meas_) is obtained by the following equation:
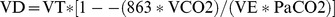
with 863 as a constant and P_a_CO_2_ as pressure for arterial CO_2_.

In healthy individuals [29], but not in HF patients [30], P_a_CO_2_ can be reliably estimated from end-tidal expiratory pressure for CO_2_ (P_ET_CO_2_). Therefore, we measured P_a_CO_2_ from arterial gas sampling in HF patients, and we estimated P_a_CO_2_ from P_ET_CO_2_ in healthy subjects. Thus, only in HF patients, a small catheter was introduced into a radial artery, blood samples were obtained at rest and every 2 minutes during exercise, and P_a_CO_2_ was determined with a pH/blood gas analyzer (GEM 4000, Instrumentation Laboratory, Bedford, MA, US).

We calculated possible VD changes during exercise, and we evaluated whether an added DS modifies the slope of the VE vs. VCO_2_ relationship and/or it simply upshifts it.

### Statistical analysis

Data are mean ± standard deviation (SD). Cardiopulmonary measurements were collected breath by breath and reported as average over 20 s. Comparisons between the two groups were done through unpaired t-test. Both in HF and in healthy subjects, analysis of variance for repeated measures with Bonferroni post hoc test was performed to analyze the effect of the adding of different DS and to evaluate the changes of VD_meas_ during exercise in the 3 experimental conditions. Bland and Altman relationship was calculated to compare VD_Yint_ values and VD_meas_ values in HF patients and in healthy individuals.

Statistical significance was set at p<0.05. All statistics were performed with IBM SPSS statistics 20.0 for windows.

## Results

We enrolled 10 HF patients (9 males; mean age 61±13 years) and 10 age-matched healthy subjects (8 males; mean age 59±10 years). The main anthropometric data were not significantly different between the two groups. Patients with HF and healthy subjects were free from obstructive defects; although within the predicted normal limits, lung volumes tended to be smaller in HF patients than in normal subjects (table 1).

**Table 1:** Main anthropometric characteristics, demographical and pulmonary function data of heart failure patients and healthy subjects enrolled in the study.

	HEART FAILURE PATIENTS	HEALTHY SUBJECTS	p value
**Number**	10	10	NS
**Male/female**	9/1	8/2	NS
**Age (yr)**	61±12	59±7	NS
**Height (cm)**	172±9	173±6	NS
**Weight (Kg)**	85±15	77±11	NS
**BMI (Kg/m^2^)**	28.6±3.8	25.4±3.2	NS
**VC (L)**	3.58±0.75	4.72±1.03	<0.01
**VC (% predicted)**	91±14	112±13	<0.01
**FVC (L)**	3.47±0.67	4.63±1.10	<0.01
**FVC (% predicted)**	90±12	112±14	<0.01
**FEV_1_ (L)**	2.56±0.58	3.57±0.84	<0.001
**FEV_1_ (% predicted)**	79±14	107±17	<0.001
**FEV_1_/FVC**	73±4	76±5	NS

Data are presented as number or mean ± SD. **BMI** =  body mass index; **NS** =  not significant; **FEV_1_** =  forced expiratory volume in 1 s; **FVC** =  forced vital capacity; **VC** =  vital capacity.

### HF patients

Mean left ventricle ejection fraction was 33±5%. The cause of HF was ischemic dilated cardiomyopathy in 4 cases and primary dilated cardiomyopathy in 6 cases. Three patients had an implantable cardioverter defibrillator; 9 were in sinus rhythm and 1 was in permanent atrial fibrillation. Four patients were in NYHA class I, 5 in NYHA class II and 1 in NYHA class III. All HF patients were on β-blockers, 9 with angiotensin-converting enzyme inhibitors, 4 with aldosterone receptor antagonists, 5 with diuretics and 3 with amiodarone.

All HF patients performed CPET without added DS and with 250 mL and 500 mL of additional DS without complications. In the HF group, peak VO_2_ was slightly reduced compared to healthy subjects. With the exception of reduced peak workload and of an increased VT, the adding of different DS did not significantly impact on CPET data at peak of exercise and on VO_2_ at AT (table 2). In table 3 VE, RR, VT, VD/VT, VCO_2_, P_ET_CO_2_ and P_a_CO_2_ during exercise are reported with 0, 250 and 500 mL of added DS.

**Table 2:** Cardiopulmonary exercise testing data in heart failure patients (upper panel) and healthy subjects (lower panel) with 0 mL, 250 mL and 500 mL of additional dead space.

HEART FAILURE PATIENTS	ADDED DEAD SPACE			ANOVA p value
	+0 mL	+250 mL	+500 mL	
**Peak workload (W)**	109±41*	103±47	96±41	0.006
**Peak VO_2_ (mL/min/Kg)**	19.9±5.8	19.3±5.6	19.6±5	NS
**VO_2_ at AT (mL/min/Kg)**	13±3	14.1±4	12.7±5.8	NS
**Peak O_2_ pulse (mL/beat)**	15.8±5.7	15.4±5.2	15.7±4.8	NS
**Peak HR (beat/min)**	111±26	110±28	104±20	NS
**Peak VT (L)**	1.9±0.49	1.93±0.49$	2.09±0.59	0.047
**Peak VE (L/min)**	55.6±14	59.8±14	58.8±11	NS
**Peak RR (bpm)**	30±4	31±5	30±5	NS
**Peak PaO_2_ (mmHg)**	107±12	104±16	100±20	NS
**Peak SaO_2_ (L/min)**	98.4±1.2	97.5±1.9	97.7±1.7	NS
**HEALTHY SUBJECTS**				
**Peak workload (W)**	200±51	195±51	189±45	NS
**Peak VO_2_ (mL/min/Kg)**	36.1±8.4	35.6±7.2	35.8±7.5	NS
**VO_2_ at AT (mL/min/Kg)**	21.7±5.7	23.6±3.7	25.3±6.6	NS
**Peak O_2_ pulse (mL/beat)**	17.5±4.2	17±2.9	18.4±3.4	NS
**Peak HR (beat/min)**	156±18	157±18	156±18	NS
**Peak VT (L)**	2.71±0.6	2.57±0.9	2.95±0.5	NS
**Peak VE (L/min)**	88.6±21.9	87.2±16.2	88.6±17.1	NS
**Peak RR (bpm)**	32±4	32±6	30±5	NS

Data are presented as means ± SD; **AT** = anaerobic threshold; **bpm** =  breaths per minute; **HR** = heart rate; **NS** =  not significant; **P_a_O_2_** =  arterial oxygen pressure; **RR** = respiratory rate; S_a_O_2_ =  arterial oxygen saturation; **RR** = respiratory rate; **VO_2_** = oxygen consumption; **VE** = ventilation; **VT** = tidal volume; **W** = watt.

$ p<0.05 versus +500 mL; * p<0.01 versus +500 mL.

**Table 3:** Ventilatory parameters in heart failure patients with 0, 250 and 500 mL of additional dead space.

HF PATIENTS	+0 mL			+250 mL			+500 mL			ANOVA p value
**Rest**										
VE (L/min)	11.8	±	1.7^$μ^	16.2	±	3.5	20.0	±	4.2	<0.001
RR (bpm)	14.2	±	2.0	16.4	±	4.1	16.8	±	3.1	NS
VT (L)	0.8	±	0.2*	1.0	±	0.2^£^	1.2	±	0.1	<0.001
VD/VT	0.47	±	0.15^$&^	0.61	±	0.10	0.67	±	0.11	<0.001
VCO_2_ (L/min)	0.25	±	0.06	0.29	±	0.13	0.29	±	0.14	NS
P_ET_CO_2_ (mmHg)	33.4	±	1.6	33.0	±	2.5	33.1	±	4.2	NS
P_a_CO_2_ (mmHg)	35.8	±	2.2^$μ^	38.6	±	1.9	39.9	±	2.02	<0.001
**4 min exercise**										
VE (L/min)	21.6	±	3.8μ^#^	30.2	±	5.0	34.8	±	4.3	<0.001
RR (bpm)	18.7	±	2.7	20.4	±	4.3	20.7	±	4.1	NS
VT (L)	1.2	±	0.2^&^	1.5	±	0.3	1.7	±	0.3	<0.001
VD/VT	0.33	±	0.09^$μ^	0.45	±	0.06	0.54	±	0.10	<0.001
VCO_2_ (L/min)	0.64	±	0.15	0.74	±	0.17	0.73	±	0.21	NS
P_ET_CO_2_ (mmHg)	37.2	±	2.9	35.7	±	3.6	37.4	±	4.2	NS
P_a_CO_2_ (mmHg)	38.4	±	2.8	38.8	±	3.4	41.2	±	3.9	NS
**8 min exercise**										
VE (L/min)	39.9	±	5.9^μ^	44.5	±	4.8^£^	52.4	±	8.4	<0.001
RR (bpm)	25.1	±	3.2	25.3	±	5.2	26.8	±	4.6	NS
VT (L)	1.6	±	0.3	1.8	±	0.4	2.0	±	0.5	NS
VD/VT	0.28	±	0.06^μ#^	0.41	±	0.07	0.46	±	0.09	<0.001
VCO_2_ (L/min)	1.28	±	0.35	1.27	±	0.29	1.34	±	0.35	NS
P_ET_CO_2_ (mmHg)	37.2	±	4.3	36.8	±	4.6	38.5	±	4.2	NS
P_a_CO_2_ (mmHg)	38.0	±	3.7	39.4	±	4.2	41.4	±	4.6	NS
**peak exercise**										
VE (L/min)	55.7	±	14.0	59.9	±	14.6	58.9	±	11.3	NS
RR (bpm)	30.3	±	4.7	31.4	±	4.0	29.8	±	5.0	NS
VT (L)	1.9	±	0.5	1.9	±	0.5	2.1	±	0.6	NS
VD/VT	0.26	±	0.11*^μ^	0.39	±	0.10	0.45	±	0.11	<0.001
VCO_2_ (L/min)	1.81	±	0.67	1.72	±	0.68	1.58	±	0.55	NS
P_ET_CO_2_ (mmHg)	35.4	±	4.5	35.64	±	4.8	39.0	±	4.9	NS
P_a_CO_2_ (mmHg)	35.8	±	3.8	38.0	±	4.2	41.3	±	5.5	0.049

Data are presented as means ± SD; **VE** = ventilation; **RR** = respiratory rate; **VT** = tidal volume; **VD** = dead space volume; **VCO_2_** =  carbon dioxide production; **P_a_CO_2_** =  arterial carbon dioxide pressure; **P_ET_CO_2_** = End-tidal carbon dioxide pressure; **bpm** =  breaths per minute;

$ : p<0.05 vs. 250 mL; ^μ^: p<0.001 vs. 500 mL;^ *^: p<0.001 vs. 250 mL;^ &^: p<0.01 vs.500 mL;^ #^<0.01 vs. 250 mL.

Values of VE_Yint_, RR_Yint_, VD_Yint_, VD_meas_ and the slope of VE vs VCO_2_ relationship in HF patients with 0 mL, 250 mL and 500 mL of additional DS are reported in table 4.

**Table 4:** Values of the slope of VE vs VCO_2_ relationship, VE_Yint_, RR_Yint_ and volume of dead space in heart failure patients (upper panel) and healthy subjects (lower panel) with 0 mL, 250 mL and 500 mL of additional dead space.

HEART FAILURE PATIENTS	ADDED DEAD SPACE			ANOVA p value
	+0 mL	+250 mL	+500 mL	
**VE/VCO_2_ slope**	27±4	28±5	29±4	0.037
**VE_Yint_ (L/min)**	4.98±1.63† §	9.69±2.91*	13.26±3.18	0.000
**RR_Yint_ (bpm)**	13±4^&$^	15±3	16±3	0.032
**VD_Yint_ (L)**	0.39±0.07^©^ §	0.61±0.12§	0.83±0.11	0.000
**VD_meas_ (L)**	0.38±0.08^©^ §	0.61±0.12§	0.80±0.09	0.000
**HEALTHY SUBJECTS**				
**VE/VCO_2_ slope**	23±3	24±4	24±4	NS
**VE_Yint_ (L/min)**	4.9±1.4† §	9.3±2.4§	13.1±3.04	0.000
**RR_Yint_ (bpm)**	14±4	14±4	14±3	NS
**VD_Yint_ (L)**	0.37±0.11^©^ §	0.68±0.15§	0.95±0.14	0.000
**VD_meas_ (L)**	0.37±0.06^©^ §	0.68±0.11*	0.94±0.1	0.000

Data are presented as means ± SD; **RR_Yint_** = respiratory rate calculated as Y intercept of RR vs VCO_2_ relationship; **VCO_2_** =  carbon dioxide production; **VD_Yint_** =  dead space volume calculated as VE_Yint_/RR_Yint_; **VD_meas_** =  dead space volume measured by P_a_CO_2_ in heart failure patients and estimated by P_ET_CO_2_ in healthy subjects; **VE** =  ventilation; **VE_Yint_** = ventilation at VCO_2_ = 0, calculated as Y intercept of VE vs VCO_2_ relationship.

† p<0.001 versus +250 mL;

§ p<0.001 versus +500 mL;

* p<0.01 versus +500 mL;

& p<0.05 versus +250 mL;

$ p<0.05 versus +500 mL;

© p<0.01 versus +250 mL.

**Figure 2:**
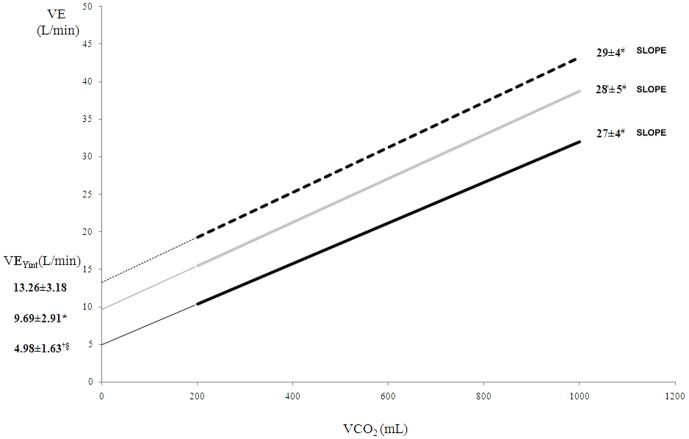
VE vs. VCO_2_ relationship in heart failure patients with 0 mL (black line), 250 mL (grey line) and 500 mL (dotted line) of additional dead space (DS). The adding of DS uplifts the VE vs. VCO_2_ relationship with a minor slope increase. † p<0.001 versus +250 mL; § p<0.001 versus +500 mL; * p<0.01 versus +500 mL;^ #^ p<0.05 versus other all.

With the adding of DS, the VE_Yint_ increased significantly, whereas RR_Yint_ showed a limited increase. Adding DS upshifted the VE vs. VCO_2_ relationship with a minor slope increase (figure 2).

The calculated VD_Yint_ rose as added DS increased; mean VD_Yint_ increase with 250 and 500 mL of added space was 226±127 mL and 446±123 mL. VD_meas_ increased during exercise in the 3 conditions albeit only as a trend when DS was not added (table 5).

**Table 5:** Values of volume of dead space at rest and during exercise in heart failure patients and healthy subjects with no additional dead space and with 250 mL and 500 mL of additional dead space.

	+0 mL						+250 mL						+500 mL					
	HF			H			HF			H			HF			H		
**VD_meas_ rest (L)**	0.38	±	0.08	0.37	±	0.06	0.61α	±	0.12	0.68^¥ μ^®	±	0.11	0.80^Ω^	±	0.09	0.94^ρ∞€£©^	±	0.10
**VD_meas_ 2′ (L)**	0.38	±	0.11	0.36	±	0.04	0.63 α	±	0.07	0.57	±	0.13	0.87	±	0.08	0.70	±	0.17
**VD_meas_ 4′ (L)**	0.39	±	0.08	0.34	±	0.05	0.68 α	±	0.11	0.56	±	0.09	0.91	±	0.09	0.67	±	0.16
**VD_meas_ 6′ (L)**	0.43	±	0.19	0.36	±	0.08	0.71	±	0.13	0.51	±	0.09	0.92	±	0.15	0.62	±	0.15
**VD_meas_ 8′ (L)**	0.43	±	0.09	0.32	±	0.08	0.73 α	±	0.11	0.48	±	0.12	0.90	±	0.14	0.57	±	0.12
**VD_meas_ peak (L)**	0.45	±	0.18	0.31	±	0.11	0.71 α	±	0.13	0.44	±	0.08	0.90	±	0.13	0.55	±	0.12
**p value**	NS			NS			0.001			0.001			0.05			0.001		

Data are presented as means ± SD; **DS** =  dead space; **H** =  healthy subjects; **HF** =  heart failure patients; **NS** =  not significant; **VD_Yint_** =  dead space volume calculated as VE_Yint_/RR_Yint_; **VD_meas_** =  dead space volume measured by P_a_CO_2_ in heart failure patients and estimated by P_ET_CO_2_ in healthy subjects.

α p<0.001 versus VD_meas_ 6′; ^Ω^ p<0.05 versus VD_meas_ 6′; ^¥^ p<0.05 versus VD_meas_ 6′; ^μ^ p< 0.001 versus VD_meas_ 8′; ® p<0.01

versus VD_meas_ peak; ^ρ^ p<0.001 versus VD_meas_ 2′; ^∞^ p<0.001 versus VD_meas_ 4′; ^€^ p<0.001 versus VD_meas_ 6′;

£ p<0.001 versus VD_meas_ 8′; ^©^ p<0.001 versus VD_meas_ peak.

**Figure 3:**
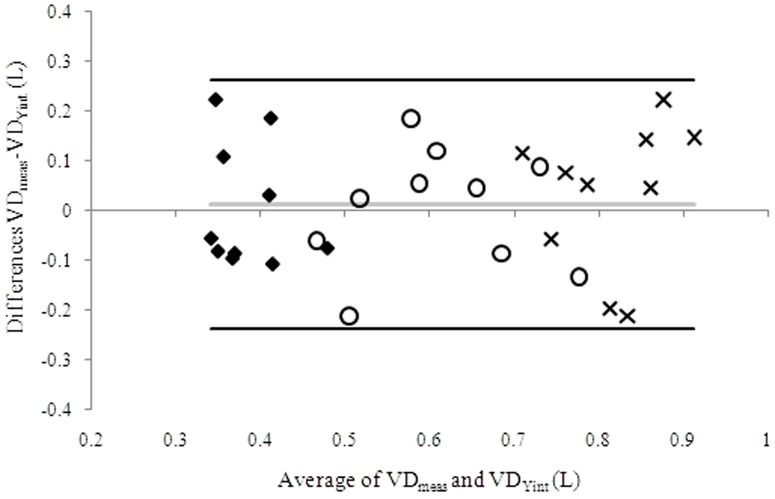
Bland and Altman plot of estimated dead space (DS) volume calculated as VE_Yint_/RR_Yint_ (VD_Yint_) and measured DS volume (VD_meas_) at rest, calculated as (1–863/P_a_CO_2_(VE/VCO_2_)*VT) for heart failure patients with 0 mL (diamonds), 250 mL (circles) and 500 mL (crosses) of additional DS. The grey line identifies the mean difference of VD_meas_ - VD_Yint;_ the black lines identify the mean difference of VD_meas_ - and VD_Yint_±1.96*standard deviation. P_a_CO_2_ =  arterial carbon dioxide pressure; VE = ventilation; VT = tidal volume.

Figure 3 reports the Bland and Altman plot of VD_Yint_ vs. VD_meas_ at rest for HF patients in the 3 exercise conditions. As an average, a good agreement was observed when VD was calculated either by VE_Yint_, or VD_meas_, with or without additional DS.

### Healthy subjects

Healthy subjects performed all CPET without complications. Peak exercise data and VO_2_ at AT were not significantly affected by the adding of DS (table 2).

**Figure 4:**
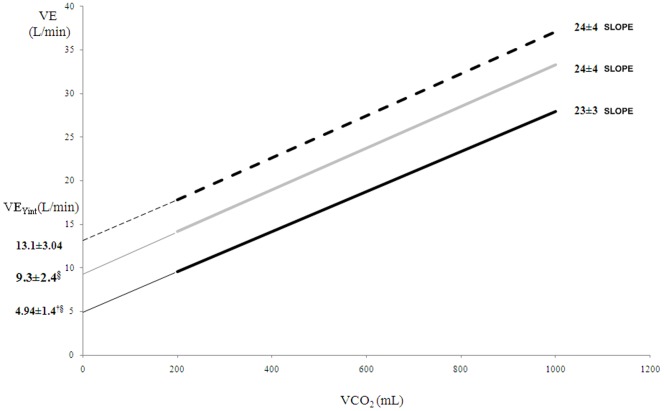
VE vs. VCO_2_ relationship in healthy subjects with 0 mL (black line), 250 mL (grey line) and 500 mL (dotted line) of additional dead space (DS). The adding of DS upshifts the VE vs VCO_2_ relationship without significant slope changes. † p<0.001 versus +250 mL; § p<0.001 versus +500 mL.

When DS was added, the value of the slope of VE vs. VCO_2_ relationship and RR_Yint_ did not change, whereas only the VE_Yint_ increased significantly (table 4) with an upshift of the relationship (figure 4). Similarly to HF patients, VD_Yint_ increased with added DS in the three experimental conditions, specifically by 300±150 mL and by 570±160 mL with 250 and 500 mL, respectively.

During exercise, VD_meas_ remained constant without additional DS, whereas it significantly decreased during exercise with added DS, but this finding is likely due to the underestimation of P_a_CO_2_ by P_ET_CO_2_ with added DS (table 5).

**Figure 5:**
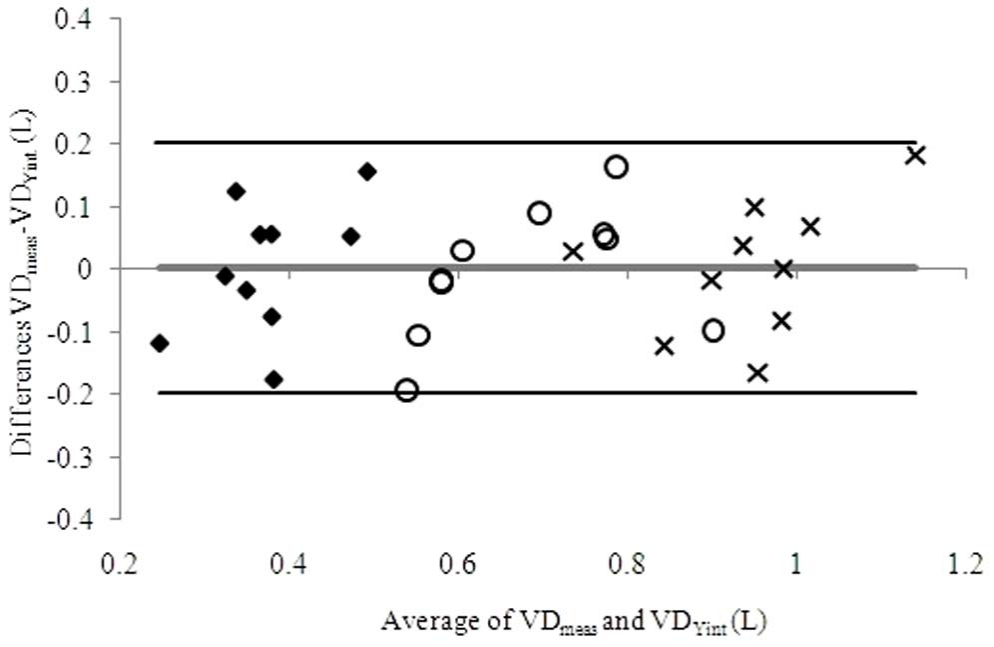
Bland and Altman plot of estimated dead space (DS) volume calculated as VE_Yint_/RR_Yint_ (VD_Yint_) and measured DS volume (VD_meas_) at rest, calculated as (1–863/P_a_CO_2_(VE/VCO_2_)*VT) with P_a_CO_2_ for healthy subjects with 0 mL (diamonds), 250 mL (circles) and 500 mL (crosses) of additional DS. The grey line identifies the mean difference of VD_meas_ - VD_Yint;_ the black lines identify the mean difference of VD_meas_ - and VD_Yint_±1.96*standard deviation. P_a_CO_2_ was estimated from P_ET_CO_2_. P_a_CO_2_ = carbon dioxide pressure; P_ET_CO_2_ =  tele-expiratory carbon dioxide pressure; VE = ventilation; VT = tidal volume.

Figure 5 reports the Bland and Altman plot of VD_Yint_ vs. VD_meas_ at rest for healthy subjects and showed a good correlation between the two methods both with and without additional DS.

## Discussion

In the present study, we evaluated a human model of increased dead space in HF patients and in healthy subjects, applying a progressive workload exercise with different added DS. We documented that a rise in serial DS, mimicking a rise in anatomical DS, was parallel to the VE_Yint_ increase both in healthy individuals and in HF patients. Therefore, VE_Yint_ is related to DS ventilation. Moreover, we showed that the value of DS can be non-invasively estimated as the ratio of VE_Yint_/RR_Yint_.

Few study limitations should be discussed at first. Firstly, our research was undertaken to analyze the role on ventilation behaviour during exercise of a respiratory comorbidity, COPD, in HF patients. We built a COPD model by adding an external dead space. We recognize that our model is only a partial COPD model because we have not considered any of the systemic consequences of COPD and we have limited our attention to DS changes. Our model was over-simplistic also as regards lung mechanics because an artificial dead space increase does not generate air trapping which is one of the most characteristic features of COPD during exercise. Secondly, our model was short lasting, so that chronic ventilatory and chemoreceptor adaptations to increased DS were not evaluated as were not evaluated primitive chemoreceptor abnormalities as drivers of the alveolar hypoventilation observed in COPD patients. Thirdly, with the Y-intercept we analyze an index of overall DS. However, in the present setting, we were able to change DS only by adding an external (anatomical equivalent) DS, so that we do not know if changes in physiological DS similarly influence the VE_Yint_. Fourthly, VE changes during exercise are due to VCO_2_, VD/VT and P_a_CO_2_ changes, and all may influence the VE vs. VCO_2_ relationship. In the present study, we added external DS, which at each step of exercise, was associated to an increase of VD/VT and P_a_CO_2_ (the latter in 2 steps only as a trend) resembling what happens during exercise in COPD patients (table 3). Therefore both P_a_CO_2_ and VD/VT changes have likely a role in the VE vs. VCO_2_ relationship changes we observed after adding DS. It is recognized that P_a_CO_2_ measurements were done only in HF patients and not in healthy subjects, but a different behaviour in healthy subjects is unlikely. Fifthly, the condition of VE at CO_2_ production equal 0, as such at the VE_Yint_ of the VE vs. VCO_2_ relationship, is a mathematical extrapolation with no physiological meaning. Moreover, absolute DS changes during exercise, so that also the VE_Yint_ value is likely close but different from the rest value. Indeed, we showed that VD tended to increase in HF patients and to reduce in healthy subjects during exercise without added DS. However, we suggest using VE_Yint_ as a tool to evaluate the presence of an increased DS, regardless of its physiological meaning with respect to rest and exercise. The adding of DS significantly reduced the external work produced in HF patients, while a not significant reduction was observed in normal subjects. Peak VO_2_ remained unchanged in both groups after adding DS; this finding suggests that added DS was associated to an increased work of breathing which, as a percentage of total work, seems to be greater in HF patients than in normal subjects.

We measured DS during exercise using a standard formula [8] in HF patients. To avoid systemic artery catheterization, we estimated P_a_CO_2_ from P_ET_CO_2_ in healthy subjects, which is a accepted method in the absence of lung disease [29]. It is recognized, however, that albeit largely used in the clinical setting, extrapolation of P_a_CO_2_ from P_ET_CO_2_ even in normal individual is approximate and likely to cause some of the variability observed (figure 5). Moreover, the values obtained in normal subjects with added DS showed a progressive and unrealistic DS reduction. This is due to a P_a_CO_2_ underestimation by P_ET_CO_2_ when adding DS, confirming the need to directly measure P_a_CO_2_ during exercise for DS evaluation [30]. The low P_ET_CO_2_ compared to P_a_CO_2_ observed during exercise with added dead space (Table 3) is likely due to the rapid rise of PCO_2_ during exhalation, which does not reach a plateau.

Adding DS increased the slope of VE vs. VCO_2_ relationship in HF patients but not in control subjects. This is different from what happens in patients with severe COPD who show a high VE/VCO_2_ ratio at the beginning of exercise but a blunted VE increase during exercise, so that the slope of VE vs. VCO_2_ relationship is normal or low [2]. In our model, the DS increase was too modest to generate a ventilatory limitation to exercise, being the ventilatory reserve at peak exercise always preserved. Accordingly, in HF patients, but not in healthy subjects, we observed a minor exercise performance reduction with the adding of DS.

The VE vs. VCO_2_ relationship is frequently used as a prognostic tool in HF patients [9]–[13]. Some laboratories prefer to analyze the ratio of the relationship [31], others the slope [32]. However, the ratio varies during exercise, so that which exercise VE/VCO_2_ ratio value should be considered is still a matter of debate [31]. Moreover, while the behaviour of VE/VCO_2_ ratio during exercise is well described in normal and HF individuals [31], its behaviour in COPD or in patients with HF and COPD is less characteristic and not used as a diagnostics/prognostic tool. To avoid the above-mentioned uncertainties, many authors prefer to study the VE vs. VCO_2_ relationship throughout the exercise [33] or up to the respiratory compensation point [8]. To do so, the slope of the VE vs. VCO_2_ relationship is calculated, but no attention is dedicated to the intercept of this relationship on the VE axis. However, the increase of the slope of VE vs. VCO_2_ relationship may be blunted when COPD is associated to HF [2]. Notably, the presence of COPD in HF may be difficult to be defined because some lung impairment is typical of HF and particularly in more advanced cases regardless of COPD [5]. In the present study, we showed that a DS increase is parallel to the VE_Yint_ increase, so that its value should be taken into account when analyzing the VE vs. VCO_2_ relationship. Indeed, VE_Yint_ differences were observed even by adding a relatively small DS (250 mL), which corresponded to 1/10 of peak VT in healthy subjects. It is recognized, however, that whilst the means of estimated and measured VD are similar, the individual values differ up to 60% in case of no added DS and up to ∼20% when 500 mL DS were added. This suggests caution when analyzing specific individual data, particularly in the presence of no or modest lung disease.

In the present study, we added 250 mL and 500 mL of DS during exercise. To confirm that VE_Yint_ increase was related to DS increase, we calculated VD_Yint_. To do so, we need to divide VE by RR, but the value of RR to be chosen is an open question. We used the intercept of the RR vs. VCO_2_ relationship on the RR axis because this is the RR value corresponding to VE_Yint_. Interestingly, the changes of VD_Yint_ values with added DS were very similar to the amount of added DS.

In conclusion, we provide the rational basis for the assessment of VE_Yint_ during exercise as a tool to evaluate DS. Further studies are needed to confirm and to analyze the clinical meaning of the present observation.

### At a Glance Commentary

The ventilation (VE) vs. VCO_2_ relationship during exercise is commonly used to assess ventilatory efficiency and prognosis in heart failure patients. The slope of the VE vs. VCO_2_ relationship increases as heart failure severity increases, whereas in respiratory patients the VE vs. VCO_2_ slope during exercise is reduced the greater the ventilatory limitation. However, respiratory disease often coexists in heart failure patients so that the mean of the slope of the VE vs. VCO_2_ relationship in these cases is unclear.

We reasoned that the VE vs. VCO_2_ behavior during exercise is a linear relationship, at least up to the respiratory compensation point, characterized by a slope and a Y intercept value. The latter has been ignored, but it represent the ventilation at VOC_2_ = 0 and therefore it is somehow related to dead space ventilation. Accordingly, we built a human model of increased anatomical dead space, resembling what happens in chronic obstructive pulmonary disease, by adding external dead space during exercise in healthy subjects and HF patients. We demonstrated that adding dead space increases the Y intercept of the VE vs. VCO_2_ relationship. The Y intercept of VE vs. VCO_2_ relationship is suggested as an index of increased dead space ventilation so that the finding of a elevated Y-intercept in a heart failure patient should bring the suspicious of a coexisting respiratory disease.
